# *Campylobacter* prevalence from food, animals, human and environmental samples in Iran: a systematic review and meta-analysis

**DOI:** 10.1186/s12866-023-02879-w

**Published:** 2023-05-10

**Authors:** Elham Ansarifar, Seyed Mohamad Riahi, Taurai Tasara, Parisa Sadighara, Tayebeh Zeinali

**Affiliations:** 1 Department of Public Health, School of Health, Social Determinants of Health Research Center, Birjand University of Medical Sciences, Birjand, Iran; 2 Cardiovascular Diseases Research Center, Birjand University of Medical Sciences, Birjand, Iran; 3 Institute for Food Safety and Hygiene, Vetsuisse Faculty, University of Zurich, Zurich, Switzerland; 4 Department of Environmental Health, Food Safety Division, School of Public Health, Tehran University of Medical Sciences, Tehran, Iran

**Keywords:** *Campylobacter*, Gastroenteritis, Meat, Feces, Milk

## Abstract

**Background:**

*Campylobacter* regarded as a major cause of foodborne gastroenteritis in humans. The present study aimed to determine the prevalence of *campylobacter* in food, animal and human samples of Iran.

**Results:**

Quantitative synthesis was performed from 119 articles. White meat had the highest pooled prevalence of *Campylobacter* spp. (43.9%). Pooled prevalence of 7.9% and 5.5% for *Campylobacter*, respectively, were determined for red meat and eggs from Iran. *Campylobacter* was seen in 14.9% of environmental samples and 8.4% of human samples. In most of the samples *C. jejuni* had higher frequency than *C. coli.* Most of the isolated *Campylobacter* harbored several of the known virulence related genes of this pathogen.

**Conclusion:**

Chicken was identified as the *Campylobacter* reservoir. As such preventive strategies in all stages of poultry production until consumption are necessary to control foodborne human infection with Campylobacter in Iran.

**Supplementary Information:**

The online version contains supplementary material available at 10.1186/s12866-023-02879-w.

## Background

*Campylobacter* species are gram-negative bacteria with different morphologies (from spiral to curved, or rod-shaped) [1]. They have single polar flagellum, bipolar flagella, or no flagellum, depending on the species. It has been reported that at least 12 species of *Campylobacter* cause human disease, the most common of which are *Campylobacter jejuni* and *Campylobacter coli* [2].

Many countries around the world recognize *C. jejuni* (~ 90%) and *C. coli* (~ 10%) as the major causative agents of human campylobacteriosis whose symptoms include diarrhea that occasionally is bloody, abdominal pain, and fever [3]. Rarely, serious long-term complications occur such as peripheral neuropathies, reactive arthritis, and Miller Fisher syndrome. Infection caused by *C. jejuni* is the most common reason of neurological sequelae [3]. *Campylobacter* is a zoonotic pathogen and its most common source is poultry [4]. In addition, contaminated water and food products, such as unpasteurized milk and contaminated fresh produce, are also known as other sources of *Campylobacter* infections [5]. *Campylobacter* infection can also occur from direct contact with infected animals, which usually carry the bacteria asymptomatically [4, 6].

According to recent data, there has been a rise in the global incidence of campylobacteriosis in most countries, although there is incomplete data from Asia, and the Middle East [7]. There is no comprehensive data on the prevalence of *Campylobacter* at the national level. This systematic review was conducted to provide comprehensive evidence on the prevalence of *Campylobacter* in human, animal, and food in Iran by using a systematic review and meta-analysis based method. Results of this study will serve as data that can be used for the prevention and control of *Campylobacter* infections in the country as well as guide to identify the research gaps.

## Results

**Fig. 1:**
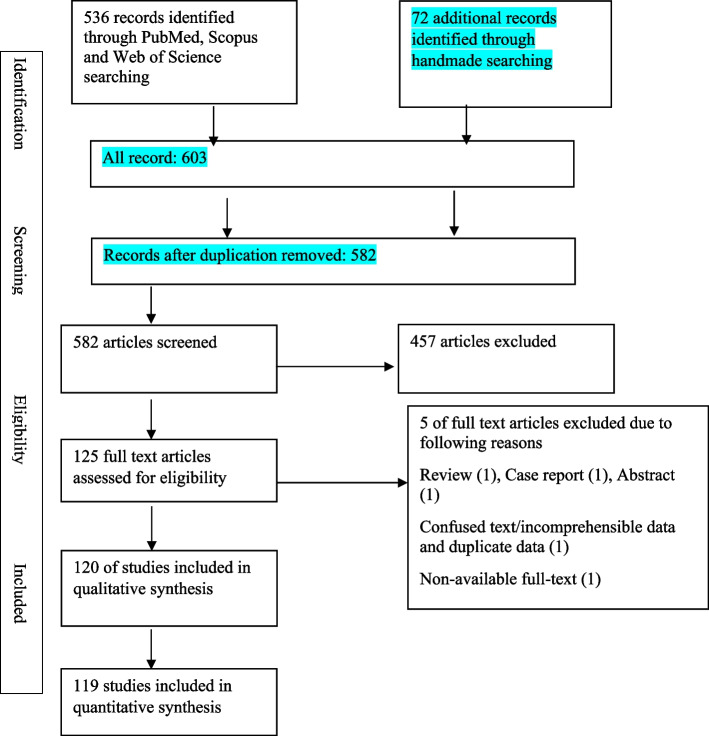
Diagram of identification and selection of studies for inclusion in the review

Overall a total of 536 articles were identified through PubMed, Scopus, and Web of Science, and 72 additional articles were identified through Google scholar, SID, and hand-based searching for the prevalence of *Campylobacter* species. Figure 1 illustrates the method applied for selecting eligible studies. 582 articles remained after removing duplicates. Based on the eligibility criteria, 457 articles were excluded. A further 5 full-text articles were excluded due to the following reasons Review (1), Case report (1), Abstract (1), confused text/incomprehensible data and duplicate data (1), Non-available full-text (1). Finally, 119 articles were included in the quantitative synthesis. Table 1 presents the detailed characteristics of every included study.

**Table 1:** Characteristics of the included study

Author	Publication year	Study years	Province	Diagnosis method	Sample source	Sample type	Sample size	Campylobacter (C. jejuni- C. coli-both)	Sample group	Place of sampling	Quality score	Reference
Abbasi, E. et al	2019	2015	Markazi	Culture + PCR	Human	Diarrhea	230	76 (76–0-0)	Feces	Hospital	10	[8]
Abdi-Hachesoo, B. etal	2014	2009	Fars	Culture + PCR	Food	Chicken	100	83 (43–40-0)	Meat	Slaughterhouse	10	[9]
Abdollahpour, N. et al	2015	2013	Razavi khorasan	Culture + PCR	Environment	Feces	200	35 (35–0-0)	Feces	Children playground	10	[10]
Akramzadeh, N. et al	2020	2018	Tehran	Culture	Food	Mechanically deboned Chicken	50	20 (0–0-0)	Meat	Farm	10	[11]
Alborzi, A. et al	2008	2003	Fars	Culture	Human	Diarrhea	243	5 (5–0-0)	Feces	Hospital	9	[12]
Ansari-Lari, M. et al	2011	2009	Fars	Culture + PCR	Animal	Cecal content	100	76 (22–32-22)	Feces	Slaughterhouse	10	[2]
Azizian, K. et al	2019	2015–2016	Kurdistan	Culture + PCR	Animal	Cecal content	200	67 (57–10-0)	Feces	Slaughterhouse	10	[13]
Divsalar, N. et al	2019	2016–2017	Mazandaran	Culture + PCR	Animal, Food, Human	Feces, Chicken, Red meat, Diarrhea	100	100 (100–0-0)	Feces, Meat	Slaughterhouse, Market, Hospital	6	[14]
Haghi, F. et al	2015	2014	Zanjan	PCR	Food	Raw milk	60	0	Product	Farm	10	[15]
Hamidian, M. et al	2011	2008–2009	Tehran	Culture + PCR	Human	Diarrhea	562	49 (34–12-0)	Feces	Hospital	9	[16]
Hassanzadeh, P. & Motamedifar, M	2007	2004	Fars	Culture	Human	Diarrhea	114	11 (11–0-0)	Feces	Hospital	10	[17]
Hoseinpour, F. et al	2017	2016	Kermanshah	PCR	Animal	Cecal content	100	55 (7–29-16)	Feces	Market	10	[18]
Jafari, F. et al	2009	2003–2005	Tehran	Culture	Human	Diarrhea	1087	60 (0–0-0)	Feces	Hospital	9	[6]
Jafari, F. et al	2008	2004–2005	Tehran	Culture	Human	Diarrhea	808	20 (0–0-0)	Feces	Hospital	10	[19]
Jahromi, R. et al	2019	2017	Fars	Culture + PCR	Food	Poultry carcass	328	223 (116–65-29)	Meat	Slaughterhouse	10	[20]
Jamshidi, A. et al	2008	2005	Razavi khorasan	Culture + PCR	Food	Poultry carcass	100	28 (0–0-0)	Meat	Slaughterhouse	10	[21]
Jonaidi-Jafari, N. et al	2016	2014–2015	Isfahan	Culture + PCR	Food	Eggshell & Egg content	440	34 (28–6-0)	Product	Market	10	[22]
Khoshbakht, R. et al	2016	2011–2013	Shiraz	Culture + PCR	Animal	Feces	302	205 (26–6-161)	Feces	Slaughterhouse	10	[23]
Khoshbakht, R. et al	2013	2011–2012	Shiraz	Culture + PCR	Animal	Feces	100	90 (48–42-0)	Feces	Slaughterhouse	10	[24]
Mahmoodipour, H. et al	2017	2016	Khuzestan	Culture + PCR	Animal	Feces	392	50 (36–14-0)	Feces	Slaughterhouse	10	[25]
Maktabi, S. et al	2019	2016	Khuzestan	Culture + PCR	Food	Chicken- Red meat	380	32 (26–6-0)	Meat	Slaughterhouse, Market	10	[26]
Malekian, M. et al	2021	2020	Isfahan	Gram stain	Environment	Feces	150	72 (-)	Feces	Landfill	9	[27]
Soltan Dallal, M.M. et al	2010	2006–2007	Tehran	Culture	Food	Chicken-Red meat	379	109 (83–26-0)	Meat	Market	10	[28]
Sharifi, S. et al	2021	2019–2020	Tehran	Culture + PCR	Human	Diarrhea	283	20 (18–2-0)	Feces	Hospital	10	[29]
Nassiri, D. et al	2016	2014	West Azerbaijan	Culture + PCR	Food	Chicken- Organ meat	552	208 (188–20-0)	Meat	Slaughterhouse	10	[30]
Nouri, S. et al	2020	2018	East Azerbaijan	Culture + PCR	Food	Organ meat	100	43 (31–12-0)	Meat	Slaughterhouse	10	[31]
Bakhshi, B. et al	2016	2012	Tehran	Culture + PCR	Food	Chicken	70	39 (0–39-0)	Meat	Market	10	[32]
Sarhangi, M. et al	2021	2018	Tehran	Culture + PCR	Human	Diarrhea	280	23 (20–3-0)	Feces	Hospital	9	[33]
Rahimi, E. et al	2017	2014–2015	Isfahan	Culture + PCR	Animal	Feces	400	28 (22–6-0)	Feces	Slaughterhouse	10	[34]
Rahimi, R. & Ameri, M	2011	2009–2010	Chaharmahal va Bakhtiari	Culture + PCR	Food	White meat	494	225 (205–20-0)	Meat	Market	10	[35]
Rahimi, E. et al	2010	2008–2009	Isfahan- Yazd	Culture	Food	Red meat	722	50 (42–8-0)	Meat	Market	10	[36]
Rahimi, E. et al	2010	2009–2010	Khuzestan	Culture + PCR	Food	White & Red meat	205	60 (53–7-0)	Meat	Market	10	[37]
Rahimi, E. et al	2010	2007–2008	Khuzestan	Culture + PCR	Food	Poultry carcass	336	213 (190–23-0)	Meat	Slaughterhouse	10	[38]
Rahimi, E. et al	2010	2007	Isfahan	PCR	Food	Poultry carcass	348	216 (175–41-0)	Meat	Slaughterhouse	10	[39]
Rahimi, E. & Tajbakhsh, E	2008	2006–2008	Isfahan	Culture	Food	White meat	800	377 (288–89-0)	Meat	Market	10	[4]
Razei, A. et al	2017	2014	Tehran	PCR	Food	Milk	30	1 (1–0-0)	Product	Market	10	[40]
Ghasemian Safaei, H. et al	2011	2008	Chaharmahal va Bakhtiari	Culture	Food	Egg	100	0	Product	Market	10	[41]
Salari, S. et al	2020	2017	Sistan va Baluchistan	PCR	Environment	Feces	100	0	Feces	Landfill	10	[42]
Torkan, S. et al	2018	2015–2016	Isfahn & Chaharmahal va Bakhtiari	Culture + PCR	Environment	Feces	100	19 (2–1-0)	Feces	Pet clinic	7	[43]
Shafiei, A. et al	2020	2018–2019	Chaharmahal va Bakhtiari	Culture + PCR	Food & Animal	Meat, liver, kidney, heart and contents of rectum	1800	126 (66–60-0)	Meat, Feces	Slaughterhouse	9	[44]
Ghane, M. et al	2011	2010	Fars	Culture	Animal,Environment	Feces	160	32 (16–9-0)	Feces	Environment	9	[45]
Ghorbanalizadgan, M. et al	2019	2018	Tehran	Culture + PCR	Human	Diarrhea	750	33 (31–2-0)	Feces	Hospital	10	[46]
Atefi Tabar, E. et al	2019	2017	Semnan	Culture + PCR	Animal	Feces	190	124 (60–0-0)	Feces	Slaughterhouse	8	[47]
Zendehbad, B. et al	2013	2012	Razavi khorasan	Culture + PCR	Food	White Meat	300	149 (127–27-0)	Meat	Market	10	[48]
Zendehbad, B. et al	2015	2013	Razavi khorasan	Culture + PCR	Food	Chicken	360	227 (200–27-0)	Meat	Market	10	[49]
Amanpour, Z. et al	2021	2018	Ilam	PCR	Human	Diarrhea	103	11 (11–0-0)	Feces	Hospital	9	[50]
Shahrokhabadi, R. et al	2013	2011–2012	Kerman	Culture + PCR	Food	Red meat	148	17 (14–3-0)	Meat	Slaughterhouse	10	[51]
Azizian, K. et al	2018	2015–2016	Kurdistan	Culture + PCR	Animal	Cecal content	200	67 (57–10-0)	Feces	Farm	10	[13]
Abbasi, E. et al	2019	2015	Markazi	Culture + PCR	Human	Diarrhea	200	5 (0–5-0)	Feces	Hospital	10	[52]
Ashrafganjooyi, S.B. & Saeide Adlei, N	2016	2008–2010	Kerman	Culture	Animal	Cecal content	600	190 (190–0-0)	Feces	Slaughterhouse	10	[53]
Dabiri, A. et al	2016	2012	Mazandaran	Culture + PCR	Food	Raw milk	72	12 (10-..-0)	Product	Collection center	10	[54]
Babaienajadbasiri, F. et al	2016	2014–2015	Alborz	Culture	Animal	Feces	150	98 (78–20-0)	Feces	Farm	10	[55]
Bagherpour, A. et al	2014	20,112,013	Khuzestan	Culture	Food	Chicken and Organ Meat	400	264 (239–25-0)	Meat	Slaughterhouse	10	[56]
Barati, M. et al	2021	2015–2017	Tehran	Culture + PCR	Human	Diarrhea	283	42 (40–2-0)	Feces	Hospital	10	[57]
Berizi, E. et al	2017	2009	Fars	Culture + PCR	Animal	Cecal content	300	180 (60–75-45)	Feces	Slaughterhouse	10	[58]
Aminshahidi, M. et al	2017	2014–2015	Fars	Culture + PCR	Human	Diarrhea	269	7 (7–0-0)	Feces	Hospital	9	[59]
Ebrahimi Lagha,F. et al	2015	2013	West Azerbaijan	Culture + PCR	Food	Organ meat	80	50 (20–20-0)	Meat	Slaughterhouse	10	[60]
Ehsannejad, F. et al	2015	2013	Tehran	Culture + PCR	Environment	Feces	660	20 (16–4-0)	Feces	Pet clinic	10	[61]
Fani, F. et al	2019	2016	Fars	Culture + PCR	Food	Chicken	90	26 (24–2-0)	Meat	Slaughterhouse	10	[62]
Jazayeri Moghadas, A. et al	2008	2007	Semnan	Culture	Human	Diarrhea	276	27 (27–0-0)	Feces	Hospital	10	[63]
Feizabadi, M.M. et al	2007	2004–2005	Tehran	Culture + PCR	Human	Diarrhea	500	35 (30–5-0)	Feces	Hospital	10	[64]
Ghane, M. et al	2011	2010	Fars	Culture	Animal	Feces	260	65 (27–18-0)	Feces	Farm	10	[65]
Ghane, M. et al	2010	2009	Mazandaran and Gilan	Culture + PCR	Environment	Feces, Water, Sewage	235	64 (21–13-0)	Feces, Environment	Farm, River, Sewage	10	[66]
Ghane, M. et al	2012	2011	Mazandaran and Gilan	Culture + PCR	Environment	Water	263	7 (7–0-0)	Environment	Caspian sea	10	[67]
Ghorbanalizadgan, M. et al	2014	2012–2013	Tehran	Culture + PCR	Human	Diarrhea	200	12 (10–2-0)	Feces	Hospital	10	[68]
Hamidian, M. et al	2011	2007–2008	Tehran	Culture + PCR	Food, Human	Red meat, Chicken, Diarrhea	798	149 (99–33-0)	Meat, Feces	Market, Hospital	10	[69]
Harzandi, N. et al	2015	2009	Alborz	PCR	Human	Diarrhea	160	18 [4–2-3)	Feces	Hospital	9	[70]
Hosseinzadeh, S. et al	2015	2011	West Azerbaijan	Culture + PCR	Food	Chicken wings	96	0	Meat	Market	10	[71]
Irajian, Gh.R. et al	2008	2007	Semnan	Culture	Human	Diarrhea	306	38 (38–0-0)	Feces	Hospital	10	[72]
Irannejhad,A. et al	2015	2014	Isfahan	Culture + PCR	Food	Chicken	160	102 (92–10-0)	Meat	Slaughterhouse	10	[73]
Jamali, H. et al	2015	2008–2010	Tehran	Culture	Animal	Cecal content	471	161 (138–23-0)	Feces	Market	10	[74]
Kafshdouzan, K. et al	2019	2015	Mazandaran	PCR	Animal	Cecal content	75	13 (11–2-0)	Feces	Urban	10	[75]
Kalantar, M. et al	2017	2012	Tehran	Culture + PCR	Food	Chicken	70	39 (0–39-0)	Meat	Market	10	[76]
Kazemeini, H. et al	2011	2008–2009	Isfahan	Culture	Food	Raw milk	120	3 (3–0-0)	Product	Farm	10	[77]
Khalili, M. & Mansouri, L	2009	2007	Kerman	Culture + PCR	Animal	Cecal content	90	3 (3–0-0)	Feces	Farm	10	[78]
Khanzadi, S. et al	2010	2009	Razavi khorasan	Culture + PCR	Food	Raw milk	200	31 (16-..-0)	Product	Bulk Tank	10	[79]
Khoshbakht, R. et al	2015	2012	Khuzestan	Culture + PCR	Environment	Feces	63	33 (17–3-0)	Feces	Wildlife refuge	10	[80]
Khosravi, A.D. et al	2011	2007–2008	Khuzestan	Culture	Human	Diarrhea	220	14 (9–5-0)	Feces	Hospital	10	[81]
Mahzouniyeh, M. et al	2013	2012	Tehran	PCR	Environment	Feces	100	39 (2–0-0)	Feces	Pet clinic	10	[82]
Modirrousta, Sh. et al	2016	2013	Zanjan	Culture	Food	Red Meat, Chicken, Eggshell	330	92 (55–29-0)	Meat, Product	Market, Farm	9	[83]
mohammadzadeh, A. et al	2012	2011	Chaharmahal va Bakhtiari	PCR	Environment	Feces	60	18 (5–0-0)	Feces	Pet clinic	10	[84]
Mokhtarian, Dalouei H. et al	2009	2008	Razavi khorasan	Culture	Food	Poultry carcass	100	31 (19–12-0)	Meat	Slaughterhouse	10	[85]
Mosallanejad, B. et al	2020	2017–2018	Khuzestan	Culture + PCR	Environment	Feces	101	37 (4–7-2)	Feces	Pet clinic	10	[86]
Negahdari, B. et al	2016	2010	Tehran	Culture + PCR	Human	Diarrhea	117	35 (27–8-0)	Feces	Hospital	9	[87]
Rahimi, E. & Torkey Baghbadorani, Z	2009	2006–2008	Isfahan	Culture	Food	Organ meat (Poultry Liver)	205	101 (85–16-0)	Meat	Market	10	[88]
Rahimi,E. et al	2013	2011	Chaharmahal va Bakhtiari	Culture + PCR	Food	Chicken & Red meat & White Meat	917	213 (193–20-0)	Meat	Market	10	[89]
Rahimi,E. et al	2008	2006–2007	Isfahan	Culture	Food	Red meat	183	0	Meat	Slaughterhouse	10	[90]
Rahimi, M.K. et al	2009	2007–2009	Tehran	Culture	Human	Diarrhea	90	7 (7–0-0)	Feces	Hospital	10	[91]
Rahimi,E. et al	2011	2009–2010	Gilan	Culture + PCR	Food	White Meat	159	52 (46–6-0)	Meat	Market	10	[92]
Rahimi,E. et al	2013	2009–2010	Isfahan- Chaharmahal va Bakhtiari	Culture + PCR	Food	Red meat	379	31 (24–7-0)	Meat	Market	10	[93]
Rahimi, E. & Esfahani, M.H	2010	2009–2010	Chaharmahal va Bakhtiari & Kohgiluyeh and Boyer-Ahmad	Culture + PCR	Food	Chicken	350	197 (183–14-0)	Meat	Market	10	[94]
Rahimi, E	2013	2010–2011	Chaharmahal va Bakhtiari	Culture + PCR	Food	Chicken & Organ meat	480	331 (301–30-0)	Meat	Slaughterhouse	10	[95]
Rashed, T. et al	1994	1993–1994	Razavi khorasan	Culture	Human	Diarrhea	903	19 (19–0-0)	Feces	Hospital	10	[96]
Ranjbar, R. Babazadeh, D	2017	2016	West Azerbaijan	Culture + PCR	Human	Diarrhea	1010	0	Feces	Hospital	10	[97]
Roshanjo, K. et al	2019	2014	Gilan	PCR	Environment	Water	45	7 (7–0-0)	Environment	River	10	[98]
Jahromi, R. et al	2021	2019	Khuzestan	Culture + PCR	Food	Poultry carcass	370	203 (130–73-0)	Meat	Slaughterhouse	10	[99]
Saadatmand. A. et al	2017	2016	Hamadan	Culture	Food	Organ meat	80	72 (53–19-0)	Meat	Market	10	[100]
Sabzmeydani, A. et al	2020	2018–2019	Mazandaran	Culture + PCR	Food	Poultry Eggshell	450	84 (45–3-0)	Product	Market	10	[101]
Sadeghi, A. et al	2020	2019–2020	Tehran	Culture + PCR	Human	Diarrhea	400	28 (24–2-0)	Feces	Hospital	10	[102]
Salehi, M. et al	2014	2011–2013	Sistan va Baluchistan	Culture	Human	Diarrhea	164	19 (19–0-0)	Feces	Hospital	10	[103]
Shahrokhabadi, R. et al	2011	2010	Kerman	Culture + PCR	Food	Chicken & Organ meat	100	31 (19–12-0)	Meat	Slaughterhouse	10	[104]
Shakerian, A	2016	2014	Chaharmahal va Bakhtiari	Culture + PCR	Food	Vegetable	100	15 (2–13-0)	Environment	Market	10	[105]
Shakerian, A. et al	2011	2006–2008	Isfahan	Culture	Food	Red meat	150	17 (13–4-0)	Meat	Slaughterhouse	10	[106]
Shams, S. et al	2017	2012–2014	Tehran	Culture + PCR	Human	Diarrhea	750	35 (33–2-0)	Feces	Hospital	9	[107]
Shirazi, M.H. et al	2013	2011	Tehran	Culture	Human	Diarrhea	117	9 (9–0-0)	Feces	Hospital	10	[108]
Soltan Dallal, M.M. et al	2016	2015	Tehran	Culture	Human	Diarrhea	305	3 (0–3-0)	Feces	Hospital	9	[109]
Taremi, M. et al	2006	2004	Tehran	Culture	Food	Red Meat & Chicken	241	88 (0–0-0)	Meat	Market	10	[110]
Tavakoli vaseksi, A. et al	2012	2010–2011	Mazandaran	Culture + PCR	Food	Raw milk	552	47 (36–11-0)	Product	Collection center	10	[111]
Zamani moghadam, A. et al	2011	2010–2011	Chaharmahal va Bakhtiari	Culture + PCR	Environment	Feces	150	1 (1–0-0)	Feces	Environment	10	[112]
Zamani moghadam, A. et al	2012	2011	Chaharmahal va Bakhtiari	Culture + PCR	Environment	Feces	120	2	Feces	Environment	10	[113]
Ziaei, N. et al	2008	2005–2006	Golestan	Culture + PCR	Human	Diarrhea	455	3 (3–0-0)	Feces	Hospital	10	[114]
Azimirad, M. et al	2021	2019	Tehran	Culture + PCR	Food	Vegetable	366	76 (24–52-0)	Environment	Market	10	[115]
Rastyani, S. et al	2015	2013–2014	Hamadan	Culture + PCR	Human	Diarrhea	120	9 (6–3-0)	Feces	Hospital	9	[3]
Raeisi, M. et al	2017	2014–2015	Mazandaran and Golestan	Culture + PCR	Food	Raw milk, Chicken, White Meat, Red meat	590	141 (79–41-0)	Product, Meat	Bulk tank, Market, Slaughterhouse	10	[5]
Rahimi, E. et al	2010	2007–2008	Isfahan	Culture	Food	Red meat	94	5 (1–4-0)	Meat	Slaughterhouse	9	[116]
Basirisalehi, M. et al	2007	2006	Fars	Culture	Animal	Feces	120	37 (15–10-0)	Feces	Farm	8	[117]
Basirisalehi, M. et al	2007	2006	Fars & Bushehr	Culture	Animal	Feces	455	85 (24–13-0)	Feces	Farm	10	[118]
Dabiri, H. et al	2014	2011–2012	Tehran	Culture	Food	Red Meat, Chicken	450	121 (93–28-0)	Meat	Market	10	[119]
Mirzaie, S. et al	2011	2010	Tehran	Culture	Animal	Cecal content	125	52 (19–33-0)	Feces	Slaughterhouse	10	[120]

### Prevalence/proportion of *Campylobacter* spp. in meat/animal products and environment of Iran

**Fig. 2:**
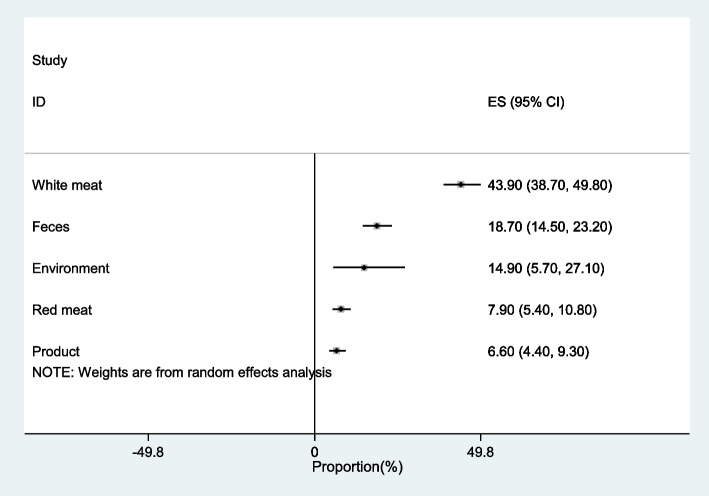
Forest plot of pooled prevalence/proportion of *Campylobacter* spp. in white and red meat, product of animal, feces and environmental samples of Iran

An overview showing the pooled *Campylobacter* spp. prevalence data generated from Iranian meat (92 studies), environment (6 studies), fecal (79 studies) and animal product sample (44 studies) categories generated using the random effects model is provided in Fig. 2. The highest prevalence of *Campylobacter* spp. has been observed in white meat (43.9%) from 55 studies among the meat and animal products that was reported in different studies from 0 to 90%. *Campylobacter* spp. prevalence in white meat was higher for chicken (48.6%) than other types of poultry meat (33.9%). Within the red meat category by 37 studies, *Campylobacter* spp. was detected at an overall pooled prevalence of 7.9% (Table 2), which was reported from 0 to 24% in the literature. *Campylobacter* contamination in this category was mostly prevalent in buffalo (13.5%), followed by goat and sheep (8.6%), cattle (8.4%) and camel (2.5%) meat. While among animal products eggs were found to have a 5.5% prevalence of *Campylobacter* spp. contamination, with a high rate of contamination prevalence being observed for chicken eggs (9.9%) in eight studies compared to eggs of other types of poultry (4.2%) from 24 studies. The prevalence of *Campylobacter* spp. contamination detected among environmental samples was 14.9%. Vegetables were constituted environmental samples that showed highest prevalence (19.4%) of *Campylobacter* contamination. Water and sewage samples had prevalence of 15.4% and 7.4%, respectively. As the I2 heterogeneity index was more than 50, there was heterogeneity in the included studies.

**Table 2:** Pooled prevalence/proportion of *Campylobacter* spp. in samples

Sample	Number of effect size	Pooled Prevalence/Proportion (%)	95% Confidence Interval	Heterogeneity (I2)
Meat	96	27.3	21.8–33.1	98.3
White meat	55	43.9	38.7–49.8	96.4
Chicken	37	48.6	41.8–55.4	96.8
Poultry	18	33.9	23.7–44.7	95.1
Red meat	37	7.9	5.4–10.8	90.6
Cattle	15	8.4	3.8–14.3	94.3
Goat-Sheep	17	8.6	5.7–11.9	83.2
Camel	3	2.5	0.7–5.3	-
Other red meat	2	13.5	7.0–21.4	-
Product	44	6.6	4.4–9.3	89
Milk	9	7.2	4.0–11.2	78.1
Egg	32	5.5	3.0–8.6	87.9
Hen	8	9.9	2.7–20.5	93.1
Poultry	24	4.2	2.0–7.0	76.9
Environment	6	14.9	5.7–27.1	-
Water	3	15.4	0.4–43.9	-
Sewage	1	7.4	0.9–24.3	-
vegetable	2	19.4	15.9–23.2	-
Feces	79	18.7	14.5–23.2	98.3
Human	34	8.4	6.0–11.1	95.8
Domestic Animal	12	21	8.2–37.6	98.1
Wild Animal	15	14.1	6.9–23.1	96.7
Poultry	18	46.8	36.4–57.3	97.0

### Prevalence/proportion of *Campylobacter* spp. in fecal samples

Literature review of 79 studies that investigated the fecal samples in animal [60] and human [34] revealed that pooled proportion of *Campylobacter* spp. was 18.7% in fecal samples. Among food animals, poultry had the highest contamination of fecal samples (46.8%). Domestic and wild animal had 21% and 14.1% contamination of *Campylobacter* spp. (Table 2). A proportion of 8.4% of human samples were positive regarding *Campylobacter* spp.

### Prevalence/proportion of *Campylobacter* spp. by place of sampling

Table 3 presents an overview from the meta-analysis of *Campylobacter* spp. prevalence from Iran based on sampling places. Poultry feces (61.9%) and white meat (47.2%) were determined to have the highest *Campylobacter* spp. prevalence at the slaughterhouse. This was followed by white meat at market (42.6%) and farm (40%) levels. The lowest pooled prevalence of *Campylobacter* spp. was observed for milk sampled at farm (1%) and market (3.3%) levels, eggs sampled at market (5.4%) and red meat sampled at slaughterhouse (6.2%) levels. *Campylobacter* spp. prevalence in white and red meat, and milk samples at markets (sampled from retails, supermarkets and butcher’s) was higher than at farms (Table 3). Considerable proportions of wild animal (prevalence of 25.4%) and dog and cat feces (prevalence of 20.4%), were found to be contaminated with *Campylobacter* spp..

**Table 3:** Pooled Prevalence/proportion of *Campylobacter* spp. by sampling place

Place	Number of effect size	Pooled Prevalence/Proportion (%)	95% Confidence Interval	Heterogeneity (I2)
**Slaughterhouse (Feces)**				
Poultry	9	61.9	44.9–77.7	97.8
Domestic animals	8	25.3	7.4–48.9	98.6
**Slaughterhouse (Meat)**				
White meat	18	47.2	37.5–57.0	97.5
Red meat	21	6.2	3.5–9.4	89.2
**Market**				
White meat	38	42.6	36.0–49.4	95.5
Poultry feces	2	37.7	33.7–48.7	-
Vegetables	2	19.4	15.9–23.2	-
Red meat	17	10.2	6.6–14.4	91.2
Egg	33	5.4	3.1–8.1	85.7
Milk	1	3.3	0.1–17.2	-
**Farm**				
White meat	1	40	26.4–54.8	-
Egg	1	31.7	23.5–40.8	-
Poultry feces	6	31.1	15.8–48.9	96.1
Wild animal feces	2	25.4	16.7–35.1	-
Domestic animal feces	4	13.5	2.2–31.2	93.0
Milk	3	1	0.01–3.5	-
Pet clinic (Dog and cat feces)	7	20.4	8.6–35.6	97.4
Hospital (Human feces)	34	8.4	6.0–11.1	95.8

### Prevalence/proportion of *C. jejuni *and* C. coli*

**Fig. 3:**
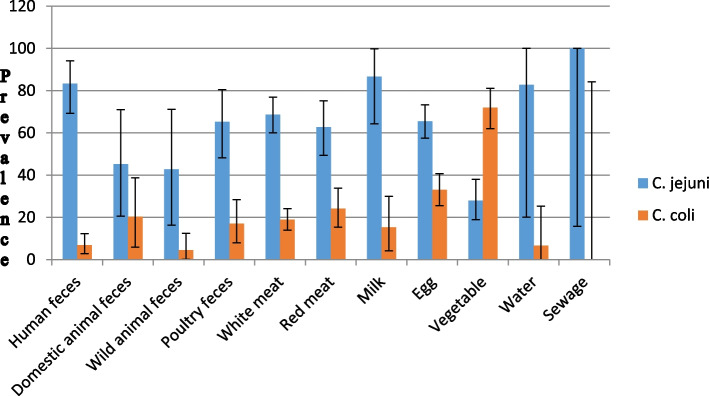
Pooled prevalence/proportion of *C. jejuni* and *C. coli* from literature in Iran based on the different categories. Error bars show the 95% confidence interval

As the *C. jejuni* and *C. coli* are the main causative agents of human campylobacteriosis, the pooled prevalence of these two species was determined in Iran samples. Most of the studies reported the prevalence of *C. jejuni* and *C. coli* in their samples. *C. jejuni* had higher pooled prevalence/proportion than *C. coli* in all of the obtained samples except for those derived from vegetables. Sewage (100%) (one study), milk (86.6%) (7 studies), human feces (83.3%) (33 studies) and water (82.8%) (3 studies) samples had the most frequent contaminations with *C. jejuni* (Fig. 3)*.* Pooled *C. jejuni* prevalence in white meat (54 studies), egg (28 studies), poultry feces (19 studies) and red meat (35 studies) was 68.7%, 65.5%, 65.2% and 62.7%, respectively. Vegetable (2 studies) samples had the least pooled prevalence of *C. jejuni* (28%). On the other hand the highest pooled prevalence of *C. coli* was reported in vegetable samples (72%) followed by egg (33%) and red meat (24.1%) samples. Pooled prevalence of *C. coli* was zero (95%CI: 0–84.2%) in sewage samples (Fig. 3).

### Pooled proportion of virulence genes in *Campylobacter* spp.

**Fig. 4:**
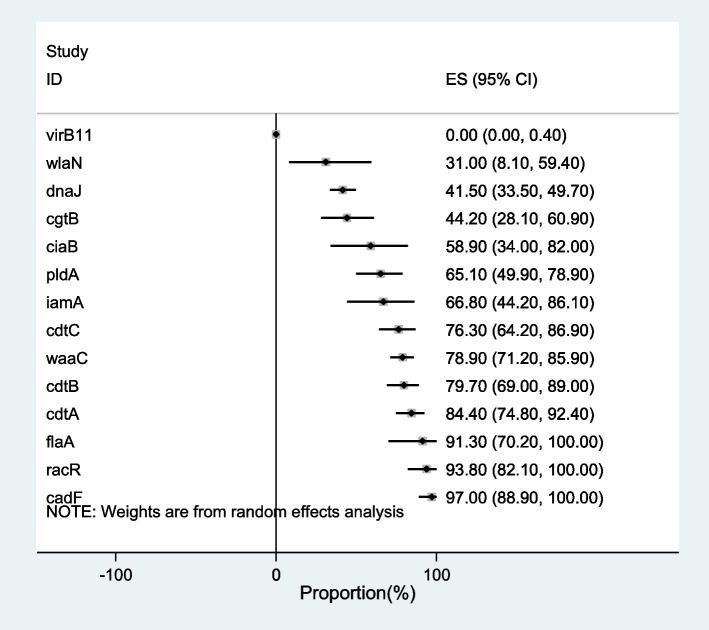
Pooled proportion of virulence genes in *Campylobacter* spp. isolates in Iran

Despite the high number of studies that reported the prevalence of *Campylobacter* spp., a limited number of them investigated the virulence genes required for pathogenesis. *CdtA, cdtB, cdtC, cadF* and *pldA* had the highest number of investigated studies. Figure 4 shows the proportion of virulence genes in *Campylobacter* spp. *cadF* (97%) had the highest pooled prevalence in *Campylobacter* spp. in 28 studies, followed by *racR* (93.8%) (3 studies) and *flaA* (91.3%) (17 studies). *VirB11* had the least prevalence (0%) in the *Campylobacter* spp. in 11 investigated studies. A total of 31% of *Campylobacter* spp. contained *wlaN* in 7 studies. With the sensitivity analysis, it was found that one of the studies pulls the results towards itself. The virB11 gene has the greatest impact on heterogeneity.

## Discussion

*Campylobacter* spp. are regarded as the commonest cause of bacterial human gastroenteritis around the world [121]. In the present study, we tried to determine the prevalence of *Campylobacter* spp. in the food, animal and human samples of Iran based on systematic review of studies published from the country. Our findings showed that in Iran, white meat including, chicken and poultry accounts for the highest pooled prevalence of *Campylobacter* spp. These results are consistent with high average *Campylobacter* contamination prevalence that has also been observed for broiler chicken (36.7%) and turkey (11.0%) meat in Europe as reported by the European Food Safety Authority [122]. *Campylobacter* spp. (33.3%) represented the second most prevalent bacterial contamination of poultry meat based on a systematic review of European surveys [123]. As much as 48.6% of chicken and 23% of other poultry meat samples in Europe were contaminated with *Campylobacter* spp. [123]. Frequency of *Campylobacter* spp. contamination in chicken was reported as 99.5% in Italy, 93.7% in Northern Ireland, 84% in Ireland, 82% in Switzerland, 56% in Turkey, 53% in Spain, 51% in Austria, 50% in Poland, 14.9% in Sweden, and 9.7% in Romania [123]. In Portugal 40.3% of fresh broiler meat samples were reported to be contaminated with *Campylobacter* spp. [124]. Our analysis in this review shows that about 76% of broiler flocks in Shiraz, Iran were positive for Campylobacter. *C. jejuni* accounted for 22% whereas *C. coli* for 32% of the Campylobacter positive chicken samples [2]. The current study revealed a higher prevalence of *C. jejuni* than *C. coli* in white meat of Iran. Poultry carcasses had 35.37% and 19.82% prevalence of *C. jejuni* and *C. coli* contaminations, respectively from the slaughterhouses of Jahrom-Iran [20]. Campylobacter was recovered from 49.2% of poultry liver, 42.8% of gizzard 33.3% of heart and 25.4% of meat from poultry slaughterhouses at West Azerbaijan, Iran [30]. The quail meat had the highest contamination (68.4%) with *Campylobacter* spp. followed by chicken (56.1%), turkey (27.4%) and ostrich meat (11.7%). The high contamination of quail meat could be due to handling in slaughtering and packaging procedure that leads to higher cross–contamination [4]. The total prevalence of *Campylobacter* spp. in poultry meat sampled from Isfahan was 47.1% [4]. Meanwhile about 55.4% of hen carcasses sampled in processing plant of Ahvaz, Iran, were contaminated with *Campylobacter* spp. [38]. Turkey samples had contamination with *Campylobacter* spp. (62.1%) [39]. Duck samples were more contaminated (39.2%) than goose samples (26.1%) [74]. Hen liver had the highest frequency of *Campylobacter* spp. (63.6%), then was turkey (40%) and ostrich liver (16.7%) [88]. Liver was more contaminated with *Campylobacter* spp. than meat [104]. Recovery of *Campylobacter* was more in chicken (63%) than beef (10%) [110]. Sheep meat (3.10%) was the most contaminated in the meat samples followed by chicken (2.40%), beef (1.80%), and buffalo meat (1.10%) from Khuzestan. 81.30% of the isolates were *C*. *jejuni* and 18.70% were *C. coli* [26]. *Campylobacter* was detected in 49.5% of chicken and 8% of beef samples [28]. Lamb meat had the highest prevalence (12%) of *Campylobacter* spp. followed by goat (9.4%), beef (2.4%) and camel meat (0.9%) [36] in Isfahan and Yazd, which was according to the present study. Higher contamination of lamb and goat meat revealed the effect of manual skinning, evisceration and processing in abattoir and inadequate hygiene in transport, storage and cutting of meat in local butcheries. Lower rate of contamination of camel milk may be related to high number of homogenic bacteria in rumen of camel and H2 accumulation that leads to destroying of campylobacter [118].

In a study that examined individual unpasteurized bovine and ovine milk samples from Zanjan, Iran, Haghi et al. [15] detected no *Campylobacter* contamination, which was in contrast to most of other studies covered in the current meta-analysis and it could be due to that other studies examined bulk milk, but Haghi et al. investigated individual milk. *Campylobacter* spp. isolated from 2.5% to 12.5% of milk samples in Mazandaran, Isfahan and Mashhad. *C. jejuni* was detected in 2.5% to 13.88% of these milk samples [5, 54, 77, 79]. Results of the current study showed 5.5% detection of *Campylobacter* spp. in eggs. Another study showed 7% contamination of eggshell of hen, 5% of duck’s eggshell, 3.3% of goose, 2.5% of ostrich, 4.2% of partridge, 5% of quail and 3.8% of turkey’s eggshell to *Campylobacter* spp. [22]. Prevalence of *C. jejuni* (6.3%) was more than *C. coli* (1.3%) in avian eggs which was according to present study. Safaei et al. [41] observed no *C. jejuni* in table eggs. 18.67% to 31.6% of eggshell were contaminated with *Campylobacter* spp. [83, 101].

Examination of cecal contents of poultry conducted in Kurdistan revealed that 55% of samples were contaminated with *Campylobacter* spp. that included *C. jejuni* (86.2%) and *C. coli* (13.7%) [13]. Similar prevalence levels have also been reported in Iran based on literature reviewed here that found *C. jejuni* is more frequent than *C. coli* in poultry feces. Khoshbakht et al. [23] reported 67.8% of *Campylobacter* spp. in cattle and sheep fecal samples of Shiraz, which was higher than current study. *C. jejuni* and *C. coli* were seen in 78.5% of the samples simultaneously. Moreover, 2.9% and 12.6% of the samples were positive for *C. coli* and *C. jejuni*, respectively [23]. Prevalence studies conducted in Isfahan detected *Campylobacter* spp. in 10%, 8%, 5.3% and 4% of sheep, goat, cattle and camel feces [34]. Salari et al. (2020) observed no *C. jejuni* in Crested lark [42]. About 33% of pet bird feces were contaminated with *Campylobacter* spp. [61]. *C. jejuni* was detected in 48.62% of bird feces [27]. 52.3% of Persian fallow deer fecal samples which were collected from Dasht-e-Arzhan located in southwest of Iran, were contaminated with *Campylobacter* spp. [80], which was higher than the present study. Most of the studies reported higher prevalence of *C. jejuni* than *C. coli* in the foodstuffs [4, 26, 28, 30, 31, 35, 36, 38, 39, 44, 51, 55, 56, 60, 83, 85, 93, 99, 101, 104] and fecal samples [13, 61, 64, 65, 70, 74, 75].

Among environmental samples examined from northern Iran, the prevalence of *Campylobacter* spp. was higher in river water (36.92%) than fecal samples of poultry (34.88%), cow (28.57%), horse (20%) and sheep (9%) origin. The lowest contaminated environmental samples were those of sewage (7.4%) origin [66]. A study that have examined Caspian Sea’s water reported a *Campylobacter* spp. contamination prevalence of 2.66% [67]. In the investigation of vegetable samples, 15% of mushrooms in Shahrekord had *Campylobacter* spp. contamination [105]. *Campylobacter* spp. was detected in 3.5% of leafy vegetables marketed in Tehran [115]. These different reported rate of contamination could be due to the difference of geographical location and season of sampling, type and number of the samples, method of isolation, and different sanitary situation on farms and slaughterhouses [49, 74].

Our current study found that human diarrheal samples examined from Iran had a pooled *Campylobacter* spp. prevalence of 8.4%. Studies from central Iran reported that 33% of infectious diarrheal samples were positive for *C. jejuni* [8]. Among acute diarrhea samples examined in Tehran, *Campylobacter* spp. were detected in 8.6% of the samples of which 69.5% were *C. jejuni* and 24.5% was *C. coli* [16]. Jafari et al., [6] studied the prevalence of *Campylobacter* spp. in children under five years of age with acute diarrhea in Tehran. They found *campylobacter* in 5.5% of patients, equal to 10.8% of all isolated bacteria. In Shiraz ~ 9.6% of acute diarrhea samples were positive for *C. jejuni* [17]. 4% of fecal samples were contaminated with *Campylobacter* spp. [46]. 9.8% of diarrheic children was positive for *C. jejuni* [63]. *C. jejuni* was the major species recovered from human samples [122].

Pathogenesis of *Campylobacter* was associated with some virulence genes. *cadF, flaA,* and *ciaB* genes are essential virulence factors for adhesion and colonization of *Campylobacter* to epithelial cells in human intestine [68]. Some studies observed 100% prevalence of *cadF* virulence gene in *C. jejuni* [14, 24, 62, 68, 76] and *C. coli* isolates [24, 68] which was agreed with the current study. The CDT toxin leads to cell cycle arrest and promotes DNA damage; so, its presence is related with the severity of the campylobacteriosis [68]. Prevalence of *cdtA, cdtB, cdtC, pldA,* and *iamA* genes were 97%, 97%, 96%, 72%, and 60%, respectively in the isolates [14], which was higher than the current study. Prevalence of *cdtA, cdtB, cdtC, racR* and *pldA* was observed 100% in some studies [24, 25, 62, 68, 69, 76]. *VirB11* gene was not detected in any of the strains [5, 24] that was according to present study and could be related to the plasmid nature of this gene [5]. Guillain–Barre’ and Miller-Fischer syndromes are associated with *wlaN, cgtB* genes and *waaC* gene [125]. Prevalence of other genes including *iamA*, and *wlaN*, was reported as 81.11%, and 82.22%, respectively [24], which was higher than current meta-analysis. Frequency of *cgtB* genes was observed as 22.22% [24] that was lower than present study. Frequency of *ciaB* was reported in 76.92% of poultry, 55.56% of cow and 100% of sheep fecal samples [25]. *pldA* and *cgtB* were detected in raw chicken *Campylobacter* isolates in Shiraz as 65.4% and 15.4%, respectively [62]. Prevalence of *dnaJ* was from 11 to 100% in different samples [69]. *WaaC* was detected in 100% of food isolates of *C. jejuni* and 75.6% of *C. coli* [5]. *Campylobacter* food isolates carried most of the virulence genes essential for pathogenesis that shows the high risk of these isolates for human.

Prevalence of *Campylobacter* spp. contamination was higher at market than farm level in Iran as determined in the present study, which is similar to observations from previous studies conducted in other countries [123]. Gonçalves-Tenório et al. [123] reported higher prevalence of *Campylobacter* spp. (44.3%) contamination at retail level than at the end-processing (30.7%) stage in poultry meat. *Campylobacter* spp. are able to colonize and attach to tissues of poultry during processing [126]. Carcass processing in the slaughterhouse including, scalding, washing and cooling was found not to decrease the level of *Campylobacter* spp. contamination of poultry meat [127]. Freezing significantly decreased chicken contamination with *Campylobacter* spp. during processing of poultry carcasses from 80 to 30% [73]. Washing reduced the contamination of sheep carcass from 10% after hiding to 8% after washing [106]. Since farms are considered as the initial site of contamination with *Campylobacter,* most preventive strategies must therefore be implemented at farm level by increasing of biosecurity and enhancing monitoring [128]. The higher contamination observed at market level may be due to uncontrolled temperature during transport of meat [5].

Poultry are regarded as a major source of this organism due to their carriage of *Campylobacter* spp. in the intestinal tract [127]. Similarly we also found here that poultry samples in Iran including meat and feces are associated with higher *Campylobacter* spp. contamination. The handling and preparation of broiler meat led to cross-contamination of poultry meat and is considered as contributing cause for one-third of human campylobacter infection in Europe while the remaining cases are related to the self-contamination of chicken with *Campylobacter* as the reservoir of the organism [122]. Establishing if such a link also exists in Iran is rather difficult due to the fact that there is currently neither notification nor investigation of food vehicles of human campylobacteriosis.

## Conclusion

In conclusion the current systematic review and meta-analysis of *Campylobacter* prevalence shows that chicken has great concern for *Campylobacter* carriage in Iran. This must be considered in preparation of undercooked poultry such as barbecue. Most of the isolated *Campylobacter* carried virulence associated genes that show their potential pathogenicity. Since our analysis showed that the gastrointestinal tract and slaughtering facilities are among the main sources of *Campylobacter* contamination for poultry meat in Iran, implementing preventive and corrective actions at several stages mainly at farm level is very vital. Implementing control strategies specifically for this pathogen will have a remarkable impact on its incidence and production of safer meat for consumers. Moreover, consumer education in hand hygiene, sanitation of surfaces prior to and after handling meat, separation of raw and cooked meat and checking the temperature of refrigerator is also needed to reduce contamination and infections with this pathogen.

## Methods

### Search strategy

A systematic search was performed in PubMed, Scopus, and Web of Science electronic databases in papers that were published from November of 2021 to the end of January 2022. The search keyword was “*Campylobacter coli* “ or “*Campylobacter jejuni*” combined with the following terms: “Food”, “Animal”, “Chicken”, “Poultry”, “Meat”, “Beef”, “Lamb”, “Fish”, “Milk”, “Dairy”, “Egg”, “Sheep”, “Goat”, “Avian”, “Cow”, “Cattle”, “Human”, “Feces”, “Diarrhea”, “Gastroenteritis “ and “Iran” (Supplementary file). Handmade search was performed in Google Scholar and scientific information database (SID). PRISMA guidelines were used to perform the systematic reviews.

### Selection criteria and quality assessment

Selection of studies were performed by these inclusion criteria: research studies including original article either published or in press; studies with a cross-sectional design to detect *Campylobacter* on the samples based on culture or PCR; had a known sample size; and studies with available full-text. Title and abstracts of the searched papers were assessed to identify articles that matched with the inclusion criteria. In some circumstances full texts were evaluated. The exclusion criteria include articles that did not follow standard methods, duplicate articles and reports, studies with unclear or incomprehensible text and analysis, articles that did not report the exact sample size and number /percent of *Campylobacter*. Positive samples Reviews; letters or editorial articles without original data were also excluded. Quality assessment of the eligible studies were performed by Joanna Briggs Institute [129]. Articles which gained 6 score (from 10) were eligible for data extraction. When two reviewers (EA and TZ) were disagreed about an article, seek the opinion of third reviewer (PS). Duplicates articles were removed by help of Endnote reference manager and also some of them were found by manual check.

### Data extraction

Data extraction forms were designed in Microsoft Excel. Articles that obtained more than 60% of quality score were eventually included in the analysis as they were meet 6 out of 10 criteria of Joanna Briggs checklist. Following information was collected from the included studies: the first author’s name, date of publication, study design, study location, number of samples, source of samples (animal, human and environment), sample group (meat, food product?, feces and environment) and type of samples (human, domestic animal, wild animal, poultry, white meat, red meat, milk, egg, water, sewage, vegetable), sample species (chicken, poultry white meat, cattle, goat, sheep, camel and other red meat, hen egg and poultry egg), place of sampling (hospital, pet clinic, slaughterhouse, farm, market and environment), diagnostic technique (Culture, PCR, culture and PCR), prevalence of *Campylobacter* spp., *C. jejuni*, *C. coli,* virulence factors and quality score.

### Statistical analysis

In this study, the data analysis was done with STATA 14 software (STATA Corp., College Station, Texas) with metaprop command. A random effect model was applied to determine the pooled prevalence and 95% Confidence interval of *Campylobacter* spp.. A forest plot was used to calculate the pooled prevalence with 95% confidence intervals. Statistical heterogeneity among studies was evaluated by computing I^2^, Cochran’s Q. 25%, 50%, and 75% of I^2^ values are classified as low, medium, and high heterogeneity, respectively. A subgroup analysis, sensitivity analysis, and meta-regression were performed on the basis of publication year, and type of sampling to evaluate sources of heterogeneity.

## Supplementary Information

**Additional file 1. **

## Data Availability

Data are available from the corresponding author on reasonable request.

## Abbreviations

PRISMA: Preferred Reporting Items for Systematic Reviews and Meta-Analyses
TZ: Tayebeh Zeinali
SMR: Seyed Mohamad Riahi
EA: Elham Ansarifar
WHO: World health organization
SD: Standard deviation
PCR: Polymerase chain reaction
Fig: Figure
