# Supplementary material for: Campylobacter prevalence from food, animals, human and environmental samples in Iran: a systematic review and meta-analysis
Source: BMC Microbiol. 2023 May 10;23:126. doi: 10.1186/s12866-023-02879-w (PMC10170724; doi:10.1186/s12866-023-02879-w)
Supplement: Supplementary file 1 — Additional file 1. [file 12866_2023_2879_MOESM1_ESM.docx]

Supplementary file: search strategy

**Pubmed**

|  | Search string | Hits |
| --- | --- | --- |
| #1 | campylobacter coli | 5,658 |
| #2 | campylobacter jejuni | 9,780 |
| #3 | chicken | 165,010 |
| #4 | poultry | 178,163 |
| #5 | poultry meat | 28,977 |
| #6 | meat | 110,599 |
| #7 | beef | 36,288 |
| #8 | lamb | 163,191 |
| #9 | fish | 312,376 |
| #10 | food | 1,344,997 |
| #11 | milk | 155,107 |
| #12 | dairy | 87,481 |
| #13 | egg | 167,715 |
| #14 | fruit* | 148,192 |
| #15 | vegetable* | #15 |
| #16 | animal | 7,178,642 |
| #17 | sheep | 148,961 |
| #18 | goat | 46,095 |
| #19 | avian | 282,273 |
| #20 | cow OR cattle | 389,199 |
| #21 | human | 20,647,355 |
| #22 | feces | 124,775 |
| #23 | diarrhea | 126,208 |
| #24 | gastroenteritis | 243,758 |
| #25 | #1 OR #2 | 11,668 |
| #26 | #3 OR #4 OR #5 OR #6 OR #7 OR #8 OR #9 OR #10 OR #11 OR #12 OR #13 OR #14 OR #15 | #26 |
| #27 | #16 OR #17 OR #18 OR #19 OR #20 | 7,218,823 |
| #28 | #21 OR #22 OR #23 OR #24 | 20,743,714 |
| #29 | Iran | 210,956 |
| #30 | #25 AND #26 | 5,166 |
| #31 | #25 AND #27 | 5,422 |
| #32 | #25 AND #28 | 8,295 |
| #33 | #30 OR #31 OR #32 | 10,399 |
| #34 | #33 AND #29 | 71 |

**Web of Science**

|  | Search string | Hits |
| --- | --- | --- |
| #1 | campylobacter coli | 1,788 |
| #2 | campylobacter jejuni | 12,090 |
| #3 | chicken | 134,180 |
| #4 | poultry | 58,313 |
| #5 | poultry meat | 3,227 |
| #6 | meat | 126,349 |
| #7 | beef | 65,377 |
| #8 | lamb | 52,699 |
| #9 | fish | 488,108 |
| #10 | food | 1,003,033 |
| #11 | milk | 236,302 |
| #12 | dairy | 139,943 |
| #13 | egg | 216,221 |
| #14 | fruit* | 341,189 |
| #15 | vegetable* | 144,129 |
| #16 | animal | 1,263,099 |
| #17 | sheep | 134,431 |
| #18 | goat | 59,700 |
| #19 | avian | 77,795 |
| #20 | cow OR cattle | 255,711 |
| #21 | human | 4,285,375 |
| #22 | feces | 36,045 |
| #23 | diarrhea | 108,962 |
| #24 | gastroenteritis | 24,640 |
| #25 | #1 OR #2 | 15,920 |
| #26 | #15 OR #14 OR #13 OR #12 OR #11 OR #10 OR #9 OR #8 OR #7 OR #6 OR #5 OR #4 OR #3 | 2,425,104 |
| #27 | #20 OR #19 OR #18 OR #17 OR #16 | 1,617,930 |
| #28 | #24 OR #23 OR #22 OR #21 | 4,413,405 |
| #29 | #26 AND #25 | 5,048 |
| #30 | #27 AND #25 | 2,218 |
| #31 | #28 AND #25 | 4,890 |
| #32 | #31 OR #30 OR #29 | 7,836 |
| #33 | Iran | 111,009 |
|  | #33 AND #32 | 56 |

**Scopus**

|  | Search string | Hits |
| --- | --- | --- |
| #1 | campylobacter coli | 3,037 |
| #2 | campylobacter jejuni | 12,665 |
| #3 | chicken | 223,943 |
| #4 | poultry | 94,433 |
| #5 | meat | 156,161 |
| #6 | beef | 58,765 |
| #7 | lamb | 53,308 |
| #8 | fish | 571,784 |
| #9 | food | 1,543,121 |
| #10 | milk | 272,863 |
| #11 | dairy | 153,969 |
| #12 | egg | 280,659 |
| #13 | fruit* | 433,660 |
| #14 | vegetable* | 243,940 |
| #15 | animal | 7,745,610 |
| #16 | human | 23,668,039 |
| #17 | feces | 193,972 |
| #18 | diarrhea | 286,616 |
| #19 | gastroenteritis | 41,728 |
| #20 | Iran | 153,844 |
| #21 | sheep | 202,483 |
| #22 | goat | 75,179 |
| #23 | avian | 98,063 |
| #24 | cow OR cattle | 500,182 |
| #25 | #1 OR #2 | 13,179 |
| #26 | #14 OR #13 OR #12 OR #11 OR #10 OR #9 OR #8 OR #7 OR #6 OR #5 OR #4 OR #3 | 8,512,812 |
| #27 | #19 OR #18 OR #17 OR #16 | 4,527,649 |
| #28 | #24 OR #23 OR #22 OR #21 OR #15 | 2,343,560 |
| #29 | #25 AND #29 | 15,077 |
| #30 | #25 AND #27 | 20,288 |
| #31 | #25 AND #26 | 16,722 |
| #32 | #31 OR #30 OR #29 | 862,136 |
| #33 | #32 AND #20 | 37,983 |
|  | Search #33 AND limit to DOCTYPE ( article OR review ) AND limit to ( AFFILCOUNTRY , "Iran" | 409 |
